# The novel AT_2_ receptor ligand, β-Pro^7^ Ang III, induces equivalent anti-fibrotic effects to Compound 21 but broader anti-fibrotic effects than pirfenidone in mice with bleomycin-induced pulmonary fibrosis

**DOI:** 10.1042/CS20245138

**Published:** 2025-07-31

**Authors:** Simon G. Royce, Cem Erdem, WeiYi Mao, Yan Wang, Mark P. Del Borgo, Robert E. Widdop, Chrishan S. Samuel

**Affiliations:** Cardiovascular Disease Program, Monash Biomedicine Discovery Institute and Department of Pharmacology, Clayton, Victoria, 3800, Australia

**Keywords:** AT_2_R, β-Pro^7 ^Ang III, interstitial collagen, pulmonary fibrosis, therapeutics

## Abstract

Angiotensin II AT2 receptor (AT_2_R) activation leads to significant anti-fibrotic and anti-inflammatory effects in diseased organs, which has led to clinical trial evaluation of the AT_2_R agonist, Compound 21 (C21), as a treatment for idiopathic pulmonary fibrosis (IPF). In this study, the anti-fibrotic effects of a more selective AT_2_R ligand, β-Pro^7^ angiotensin III (β-Pro^7^ Ang III), with >20,000-fold affinity for the AT_2_R over the AT_1_R, were compared with that of C21 or the currently used IPF medication, pirfenidone, in mice with bleomycin (BLM)-induced pulmonary fibrosis. Adult female BALB/c mice received a double intranasal instillation of BLM (20 mg/kg/day) seven days apart and were maintained until day 35, while control mice were instilled with saline (SAL) seven days apart and maintained for the same time period. Sub-groups of BLM-injured mice were then treated on day 28 with vehicle (SAL), C21 (0.3 mg/kg/day) or β-Pro^7^ Ang III (0.1 mg/kg/day) via seven-day subcutaneously implanted osmotic minipumps, or daily from days 28 to 35 via orally administered pirfenidone (100 mg/kg/day). At day-35 post-injury, measures of lung fibrosis and compliance were evaluated. Compared with their SAL-instilled counterparts, SAL-treated BLM-injured mice presented with a significantly increased lung Ashcroft score, Masson’s trichrome-stained and second harmonics generation-measured fibrosis, myofibroblast accumulation, and TGF-β1 expression, but reduced lung dynamic compliance at day-35 post-injury. While all treatments evaluated attenuated the BLM-induced lung myofibroblast accumulation and TGF-β1 expression, AT2R stimulation, but not pirfenidone, attenuated lung collagen deposition after seven days. β-Pro^7^ Ang III also significantly restored lung compliance and promoted collagen-degrading matrix metalloproteinase-2 activity. These findings highlighted the therapeutic value of selectively targeting the AT_2_R for treating IPF.

## Introduction

Idiopathic pulmonary fibrosis (IPF) is a restrictive lung disease with an unknown etiology, characterized by diminished lung volume and an irreversible decline in lung function, due to the progressive thickening and build-up of interstitial lung fibrosis [1–3]. IPF affects 1–20 per 100,000 people around the world, particularly in Western countries where it is more prevalent in people over the age of 65 years [4,5]. It has a mortality rate of ~35–40% within three years and ~50–55% within five years of diagnosis, with women tending to have a slightly longer median survival time (3.9 years) compared with men (2.8 years) [6]. There are three stages of IPF, the first of which involves factors that predispose susceptibility to IPF, such as genetic factors, aging, pulmonary hypertension, and/or exposure to tobacco smoke or other irritants and pollutants [7,8]. The second stage involves activation of the immune system and the inflammatory response to lung injury, which results in an influx of immune cells to the site of injury, the secretion of various pro-inflammatory and pro-fibrotic factors, and the triggering of epithelial-to-mesenchymal cell transition to facilitate wound healing [9]. Most notably, macrophages, epithelial cells, and/or collagen-producing fibroblasts secrete transforming growth factor (TGF)-β1, which plays several key roles in contributing to IPF pathology when overactivated [9,10]. The third stage involves the differentiation of fibroblasts into activated myofibroblasts and the remodeling and build-up of extracellular matrix (ECM) components, primarily collagen, which leads to lung stiffness and dysfunction [9,11].

Despite the pathological consequences of IPF, there are currently no effective cures for its regression. Two currently available anti-fibrotic drugs, pirfenidone, which blocks the pro-fibrotic actions of TGF-β1 and related growth factors [12], and nintedanib, which inhibits tyrosine kinase receptor activity to suppress the actions of various growth factor receptors, such as fibroblast growth factor receptor, vascular endothelial growth factor receptor, and platelet-derived growth factor receptor [13], only suppress the pro-fibrotic contributions of these growth factors and related receptors to myofibroblast differentiation and ECM/collagen production. However, these drugs do not resolve established fibrosis and can be associated with several side effects when chronically administered [14], which may lead to the discontinuation of these treatments. Hence, there is an urgent need for an alternative therapeutic option that can particularly regress established disease pathology.

The angiotensin II AT2 receptor (AT_2_R) has emerged as a suitable therapeutic target that is up-regulated in diseased settings, including IPF [15], to confer organ protection via anti-inflammatory, anti-fibrotic, anti-proliferative, anti-oxidant, and vasodilatory effects [16–19]. The AT_2_R is a G protein-coupled receptor (GPCR) that is expressed on epithelial cells [20], macrophages, and (myo)fibroblasts [21,22] within the lung. Unlike other GPCRs, however, the AT_2_R does not undergo desensitization or degradation [23], which allows for its prolonged activity [24]. This has led to the small molecule AT_2_R agonist, Compound 21 (C21), being evaluated for its therapeutic effects in preclinical models of bleomycin (BLM)-induced pulmonary fibrosis [25] and pulmonary hypertension [26], as well as in clinical trials involving patients with either IPF (Clinical Trial ID: NCT04533022), COVID-19 [27] or systemic sclerosis [28]. However, a potential limitation to targeting the AT_2_R is that until recently, a very limited number of AT_2_R-selective ligands have been developed [19].

To overcome this, numerous highly selective AT_2_R agonists were recently developed by substituting each individual amino acid within the native angiotensin II [29] or angiotensin III (Ang III) [30] structure, which can both activate the AT_2_R, with a synthetically derived β-amino acid. β-Amino acids are similar to the naturally occurring α-amino acids, except that there is an extra carbon in the amino acid backbone of the former [31]. This subtle chemical modification to native peptides can cause conformational changes that may alter their binding affinity and biological stability. From these newly developed peptide mimetics, β-Pro^7^ angiotensin III (β-Pro^7^ Ang III) was found to have >20,000 fold selectivity for the AT_2_R over the angiotensin II AT1 receptor (AT_1_R) [30], and promote the vasoprotective [30], vasodilatory [32], anti-inflammatory, and anti-fibrotic [33–35] effects of AT_2_R stimulation in the heart and kidney of preclinical models of disease. However, the extent to which β-Pro^7^ Ang III can protect the injured lung from established fibrosis is yet to be determined.

In this study, we compared the anti-fibrotic effects of β-Pro^7^ Ang III to that of C21 and the currently used IPF medication, pirfenidone, in a preclinical model of BLM-induced fibrosis, the most widely used model for evaluating novel treatments for IPF [36]. The effects of these treatments on dynamic lung compliance, as a measure of lung stiffness, were also assessed.

## Materials and methods

### Materials

BLM sulfate was obtained from Cayman Chemical (catalogue number: 9041–93-4; Ann Arbor, MI, U.S.A.). C21 was kindly provided by Vicore Pharma (Gothenburg, Sweden). Pirfenidone was purchased from MedChemExpress (Monmouth Junction, NJ, U.S.A.).

### Animals and ethics

Eight–ten-week-old female BALB/c mice, weighing approximately 20–25 grams, were obtained from Monash University Animal Services (Clayton, Victoria, Australia). All mice were housed under a controlled environment, given a five–six-day acclimatization period before any experiments were conducted on them, and maintained on a 12-hour light:12-hour dark light cycle with free access to normal rodent lab chow (Barastock Stockfeeds, Pakenham, Victoria, Australia) and water. All experimental procedures performed were approved by an Animal Ethics Committee of Monash University (MARP/2018/15167), which adheres to the National Institutes of Health (NIH) Guidelines for the Care and Use of Laboratory Animals for Scientific Purposes. Female mice were used, as they had been shown to have larger lung volumes and corresponding airway reactivity compared with their male counterparts [37]. Although BALB/c mice were found to be somewhat resistant to BLM-induced injury compared with other strains of mice [38], our previous studies had determined that female BALB/c mice underwent a significant increase in lung inflammation and fibrosis, and a loss of lung dynamic compliance by 21-day post-BLM-induced injury, when BLM was twice intranasally (i.n.)-instilled into these mice, seven days apart [39].

### Induction and treatment of BLM-induced pulmonary fibrosis

Female mice (*n*=32) were i.n.‐instilled with BLM on days 0 and 7 (0.15 mg in 50 μl of saline (SAL); 25 μl per nare on each day) [40], and then left for a further four weeks (from the second administration of BLM) for lung inflammation and fibrosis to develop. At day-28 post-injury, sub-groups of BLM-injured mice were anesthetized with inhaled isoflurane (2–3% in oxygen; Baxter Healthcare; Brunswick, Victoria, Australia) and subcutaneously implanted with osmotic minipumps (Model 1007D, with a flow rate of 0.5 μl per hour; Alzet, Cupertino, CA, U.S.A.) that contained either SAL (*n*=8), C21 (0.3 mg/kg/day; a dose typically used to induce its therapeutic effects [41,42]; *n*=8) or β-Pro^7^ Ang III (0.1 mg/kg/day; a dose used to induce its cardio- and renoprotective effects [33–35]). These minipumps were maintained for a seven-day period, until day-35 post-injury. The fourth sub-group of BLM-injured mice (*n* = 8) was twice-daily administered with pirfenidone (100 mg/kg/day; a dose typically used to induce its anti-fibrotic effects [39,43]), from days 28–35 via oral gavage. A further control group of mice was i.n.‐administered 50 μl of SAL on days 0 and 7 (25 μl per nare on each day) and maintained until day 35 (healthy control group; *n*=8).

To account for the possibility that some mice might fail to tolerate BLM or undergo a BLM-induced loss of body weight (by >20%), power calculations were performed to ensure that adequate group sizes were used for the studies detailed below; where it was determined that with a 25% standard deviation, we would be 80% powered to detect a 25–30% effect with *n*=6–8 animals per group.

### Evaluation of dynamic lung compliance

On day-35 post-BLM or SAL administration to mice, all mice were evaluated by plethysmography for changes in dynamic lung compliance [39,44]. Mice were briefly anesthetized with an intraperitoneal (i.p) injection of ketamine (100 mg/kg body weight) and xylazine (20 mg/kg body weight), tracheostomized, and cannulated. As bronchial smooth muscle contraction has been found to significantly influence lung compliance [45], methacholine (50 mg/mL) was nebulized, and dynamic compliance was measured (Biosystem XA version 2.7.9, Buxco Electronics, Troy, NY, U.S.A.) for 2 min after methacholine. To illustrate changes in dynamic compliance between the various treatment groups, the respective % change in dynamic compliance from each respective baseline in response to methacholine was calculated.

### Tissue collection

Once dynamic lung measurements were completed, anesthetized mice were humanely killed by removal of the heart before their lung tissue was collected. The lungs of each animal were then divided along the transverse plane, resulting in four separate lobes. In each case, the whole left lung lobe was fixed in 10% neutral buffered formalin overnight and processed to be cut (transversely across the length of the lobe to maximize sampling of cranial, mid, and caudal lung) and embedded in paraffin wax. The second largest lobe was used for protein extraction and the analysis of matrix metalloproteinase (MMP; gelatinase) activity. The remaining lobes were separately snap‐frozen in liquid nitrogen and eventually stored at −80°C until required.

### Histology and immunohistochemistry

Serial paraffin-embedded lung sections were processed and stained with Masson’s trichrome for the scoring of fibrosis, either as a percentage of the total area analyzed or from using the Ashcroft scale (from 0 to 8) [46].

Immunohistochemical (IHC) staining was also performed in separate serial paraffin sections, which were used to assess the localization of interstitial TGF-β_1_ or α-smooth muscle actin (SMA; as a marker of myofibroblast differentiation) expression within the lung. All sections underwent antigen retrieval in a sodium citrate buffer and incubation with antibody diluent (Dako, North Sydney, New South Wales, Australia) to block non-specific immunoreactivity, and then incubated overnight at 4°C with either a rabbit polyclonal IgG antibody to detect TGF-β1 (#ab92486, 1:500 dilution; Abcam Antibodies) or a mouse monoclonal IgG2a antibody, clone 1A4 to detect α-SMA (#M0851, 1:1000 dilution; Dako). On the following day, sections were either stained with a Dako Envision + streptavidin-horse radish peroxidase-conjugated anti-mouse (#E0464; Agilent Technologies, Mulgrave, Victoria, Australia; for the detection of α-SMA) or anti-rabbit (#K4003; Agilent Technologies; for the detection of TGF-β_1_) secondary antibodies. 3,3′-diaminobenzidine (Dako) was then added to sections for signal detection. Sections were counterstained with hematoxylin and mounted in DePex (VWR International, Radnor, PA, U.S.A.).

All Masson’s trichrome- and IHC-stained sections were scanned and captured with the Aperio Scanscope AT Turbo (Leica Microsystems Pty Ltd, VIC, Australia) and assessed with ImageJ 1.48 software (NIH, Bethesda, MD, U.S.A.). Morphometric analysis was randomly performed on six–eight consecutive non-overlapping fields per lung section (at ×400 magnification) that were void of large airways and blood vessels, by a blinded investigator. Ashcroft scoring was performed using the eight-point modified grading scale [46], while interstitial ECM deposition (fibrosis), α-SMA staining, and TGF-β1 staining were all expressed as the mean % stained area relative to the total area of the field analyzed [39] for each sample per group analyzed.

### Second harmonics generation analysis of fibrosis

A separate serial lung section from each animal was dewaxed for assessment using the HistoIndex second harmonics generation (SHG)-based platform, as previously performed on liver [47] and kidney [48] sections from murine models of disease. The HistoIndex platform detected SHG-associated interstitial (type I and III) collagen deposition within scanned tissue sections, and two-photon excitation fluorescence signals of non-collagenous tissue, respectively [49]. However, to prevent the laser-induced damage of non-collagenous tissue, several steps were taken to optimize the SHG imaging of lung sections, as outlined previously [48]. Using the FibroIndex analysis software, the % collagen area overlapping the tissue area analyzed was measured.

### Gelatin zymography

Total protein was extracted from the second largest lung lobe of each mouse using a previously outlined procedure [50] and used to assess the activity of collagen-degrading MMP-2 (gelatinase A) and MMP-9 (gelatinase B) activity by gelatin zymography. Equal amounts of tissue extracts (containing 10 µg of protein per sample) were loaded on 7.5% acrylamide gels containing 1 mg/ml gelatin and analyzed as described before [22,48]. Gelatinolytic activity was visualized as clear bands, and the optical density (OD; in arbitrary units) of each band was quantified by densitometry using a GS710 Calibrated Imaging Densitometer (Bio-Rad Laboratories, Gladesville, NSW, Australia). The mean ± standard error of the mean (SEM) OD active (A) MMP-2 or MMP-9 was then graphed and expressed as the fold mean of the value measured in the SAL-instilled control group, which was expressed as 1.

### Statistical analysis

Unless otherwise stated, all data are expressed as the mean ± SEM and were statistically analyzed with GraphPad Prism v 9.0 (GraphPad Software Inc, San Diego, CA, U.S.A.). Most of the data were analyzed using a one-way ANOVA and Tukey’s post-hoc test to allow for multiple comparisons between groups. Non-parametric (Kruskal-Wallis) tests were conducted for data that were graded based on a scoring system (i.e. Ashcroft score) or normalized to the control group (i.e. relative OD A-MMP-2 or A-MMP-9). In each case, data were considered statistically significant with a *P* value less than 0.05.

## Results

### Final animal numbers

A double administration of BLM alone, seven days apart, did not affect the mortality of mice at the dose administered. Hence, *n*=8 BLM alone injured mice and *n*=8 SAL control mice completed the five-week protocol, and lung tissues from these mice were available for analysis. However, *n*=1 BLM-injured mice treated with C21 and *n*=2 BLM-injured mice treated with pirfenidone underwent a BLM-induced loss of body weight (by >20%; after receiving the double instillation of BLM and between weeks 2–3; Table 1), which was the trigger point (as determined by the Monash Animal Ethics Committee) for these mice to be humanely killed prior to completion of the 35-day experimental period. As a result of this, lung tissues from *n*=7 BLM-injured mice treated with C21, *n*=8 BLM-injured mice treated with β-Pro^7^ Ang III, and *n*=6 BLM-injured mice treated with pirfenidone were available for analysis.

**Table 1:** The body weights of mice over the 5-week experimental period.

Groups	Saline (g)	BLM (g)	BLM + C**21** (g)	BLM+ β-Pro^7^ Ang III (g)	BLM+ pirfenidone (g)
Week-1	23.8±0.5 (8)	23.9±1.0 (8)	24.5±0.7 (8)	23.9±0.5 (8)	23.7±0.7 (8)
Week-2	24.4±0.4 (8)	21.8±0.9 (8)	22.7±0.6 (8)	22.8±0.6 (8)	22.3±0.5 (8)
Week-3	25.2±0.5 (8)	21.7±0.6^2^ (8)	22.5±0.4^2^ (8)	21.5±0.4^2^ (8)	21.4±0.6^2^ (7)
Week-4	25.5±0.6 (8)	22.2±0.7^1^ (8)	21.8±0.5^2^ (7)	20.7±0.5^2^ (8)	21.0±0.5^2^ (6)
Week-5	26.3±0.6 (8)	22.3±0.5^2^ (8)	22.0±0.8^1^ (7)	21.4±0.6^2^ (8)	21.7±0.4^2^ (6)

The mean ± SEM body weight (BW) of mice at each week outlined, as measured prior to saline or BLM instillation (at week-1 and week-2) and prior to any of the treatments administered (at week-4). One BLM-injured mouse treated with C21 was measured to have lost >20% BW at week-3 and had to be immediately killed. One BLM-injured mouse treated with pirfenidone had lost >20% BW between week-2 and week-3, while a second BLM-injured mouse treated with pirfenidone had lost >20% BW at week-3, and these mice had to be killed at those time points. Numbers in parentheses represent the numbers of mice per group measured at each time point.

1 *P*<0.05.

2 *P*<0.01 vs corresponding saline-instilled control group weights.

BW, body weight.

### β-Pro^7^ Ang III or C21, but not pirfenidone, attenuated the BLM-induced interstitial lung fibrosis after seven days of treatment

**Figure 1:**
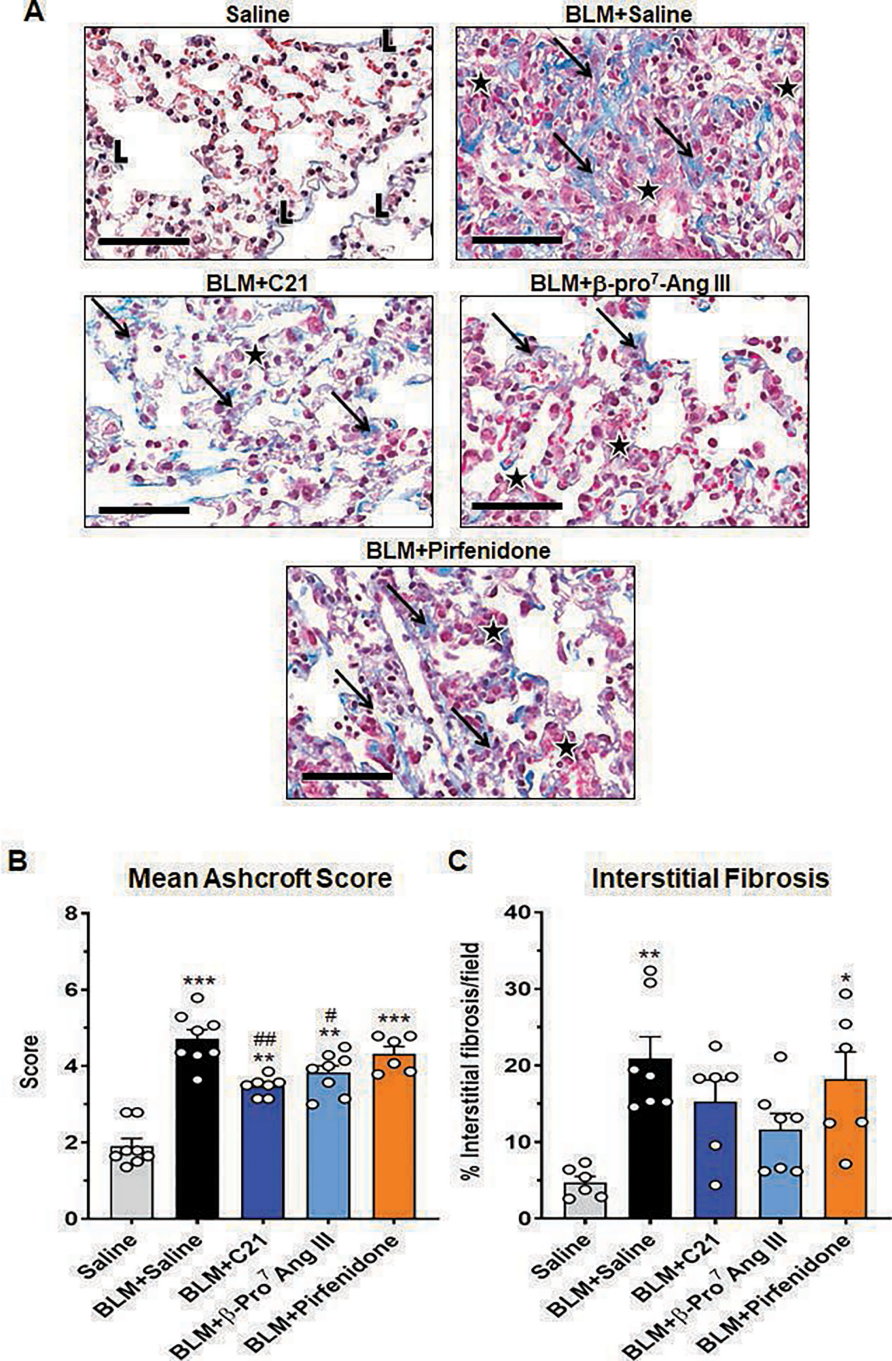
β-Pro^7^ Ang III or C21, but not pirfenidone, attenuated the BLM-induced interstitial lung fibrosis after seven days of treatment. Representative Masson’s trichrome-stained images (at ×400 magnification) show the extent of interstitial ECM accumulation (blue staining) within the lung of each of the groups investigated (**A**). Scale bar=60 μm. ‘L’ represents structural collagen; the black arrows refer to pathological collagen accumulation; and stars represent areas of cuboidal epithelial cell hypertrophy and metaplasia. Also shown are the mean ± SEM% Ashcroft score (**B**) or % interstitial fibrosis per field analysed (**C**); from 6 to 8 non-overlapping fields of view per lung section analyzed; from *n*=6–8 mice per group. The white circles represent the individual data points for each group analyzed. *P< 0.05, **P< 0.01, ***P< 0.001 vs the saline-instilled control group; ^#^ *P*< 0.05, ^##^ *P*< 0.01 vs the BLM + saline-treated group.

BLM-injured mice treated with SAL presented with significantly increased lung congestion and fibrosis at day-35 post-injury (Figure 1A), as determined by a ~1.5-fold increase in lung Ashcroft score (4.71±0.24; Figure 1B) and ~3.5-fold increase in % interstitial lung fibrosis per field analyzed (20.91±2.66%; Figure 1C) at day-35 post-injury, compared with respective measurements from their SAL-instilled counterparts (Ashcroft score: 1.91±0.20; % interstitial fibrosis per field analyzed: 4.72±0.70%; both *P*< 0.01 vs SAL-instilled counterparts). After seven days of treatment, the BLM-induced increase in lung Ashcroft score was partially, but significantly, attenuated by either C21 (3.47±0.10) or β-Pro^7^ Ang III (3.82±0.19), by 32–44% (both *P*< 0.05 vs BLM + SAL-treated group; both *P*< 0.01 vs SAL-instilled group), but not by pirfenidone (4.32±0.19; *P*< 0.001 vs SAL-instilled group; no different to BLM + SAL-treated group; Figure 1B). Similarly, after the same treatment period, the BLM-induced increase in interstitial lung fibrosis was variably blunted by C21 (15.28±2.58%) or β-Pro^7^ Ang III (11.61±1.99%), to levels that were no longer different from that measured from BLM + SAL-treated or SAL-instilled mice, but not by pirfenidone treatment (18.21±3.56%; *P*<0.05 vs SAL-instilled group; no different to BLM + SAL-treated group; Figure 1C).

**Figure 2:**
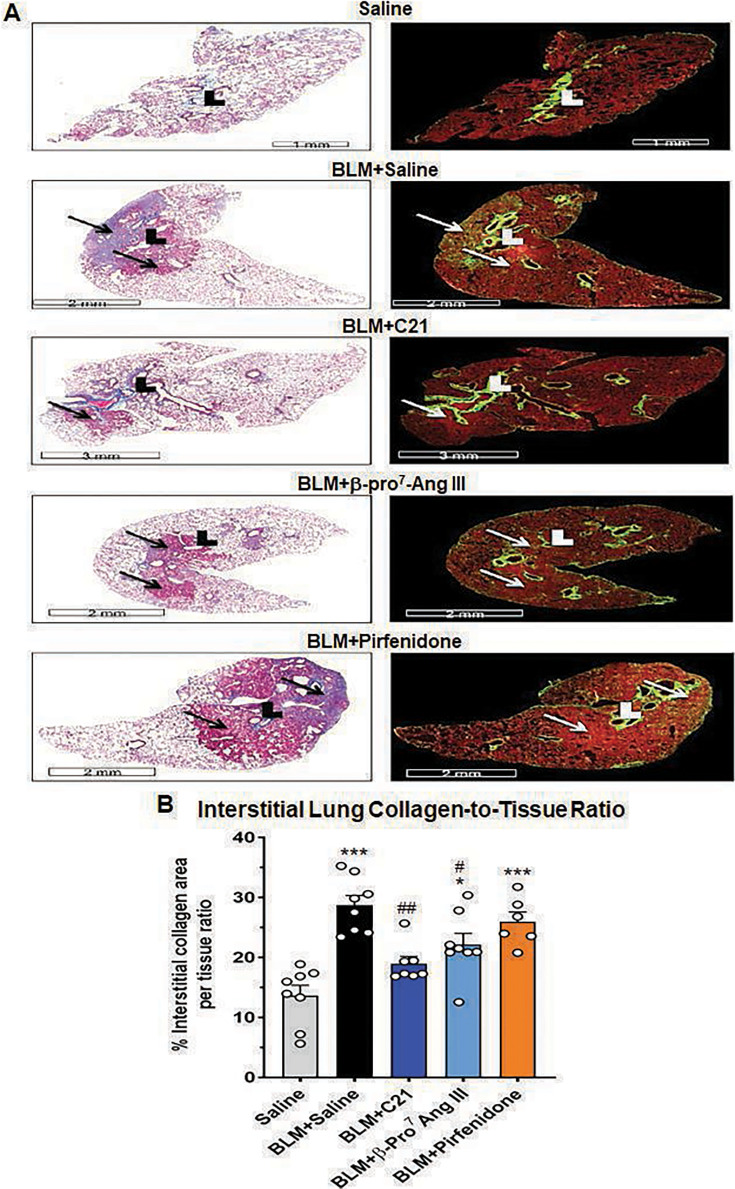
β-Pro^7^ Ang III or C21, but not pirfenidone, attenuated the BLM-induced interstitial lung collagen-to-tissue ratio after seven days of treatment. Representative serial sections from each group evaluated were stained with Masson’s trichrome (**A**) (on the left) or scanned with the HistoIndex platform (on the right). Also shown is the mean FibroIndex analyzed % SHG-detected lung collagen-to-tissue area (ratio) from each group analyzed (**B**); from *n*=6–8 mice per group. The white circles represent the individual data points for each group analyzed.*P<0.05, ***P<0.001 vs the saline-instilled control group; ^#^ *P*<0.05, ^##^ *P* < 0.01 vs the BLM + saline-treated group.

HistoIndex analysis of serial lung sections revealed a marked overlap between the SHG (green)-detected interstitial collagen and the ECM (blue-staining) detected by Masson’s trichrome (Figure 2A), indicating that most of the Masson’s trichrome-stained ECM deposition was interstitial collagen. As the SHG analysis of lung sections also detected perivascular collagen deposition (even in SAL-instilled control mice), non-overlapping regions across the interstitial space were morphometrically assessed for changes in interstitial collagen deposition rather than analyzing whole tissue sections. Based on this, it was determined that BLM-injured mice treated with SAL presented with a significantly increased SHG-based interstitial collagen area-to-tissue area ratio (28.71±1.61%) at day-35 post-injury, compared with that measured in SAL-instilled control mice (13.65±1.71%; Figure 2B). After seven days of treatment, this BLM-induced increase in interstitial collagen area-to-tissue area ratio was significantly attenuated by C21 (18.98±1.98%; *P*< 0.01 vs BLM + SAL-treated group; no different to the SAL-instilled group) or β-Pro^7^ Ang III (22.13±1.88%; *P*< 0.05 vs BLM + SAL-treated group; *P*< 0.05 vs the SAL-instilled group), but not by pirfenidone (25.95±1.62%; *P*< 0.001 vs SAL-instilled group; no different to BLM + SAL-treated group; Figure 2B). Notably, there were no significant differences between the anti-fibrotic effects of C21 versus β-Pro^7^-Ang III, on any of the measures of fibrosis assessed.

### All treatments attenuated the BLM-induced interstitial lung myofibroblast accumulation and TGF-β1 expression after seven days of treatment

**Figure 3:**
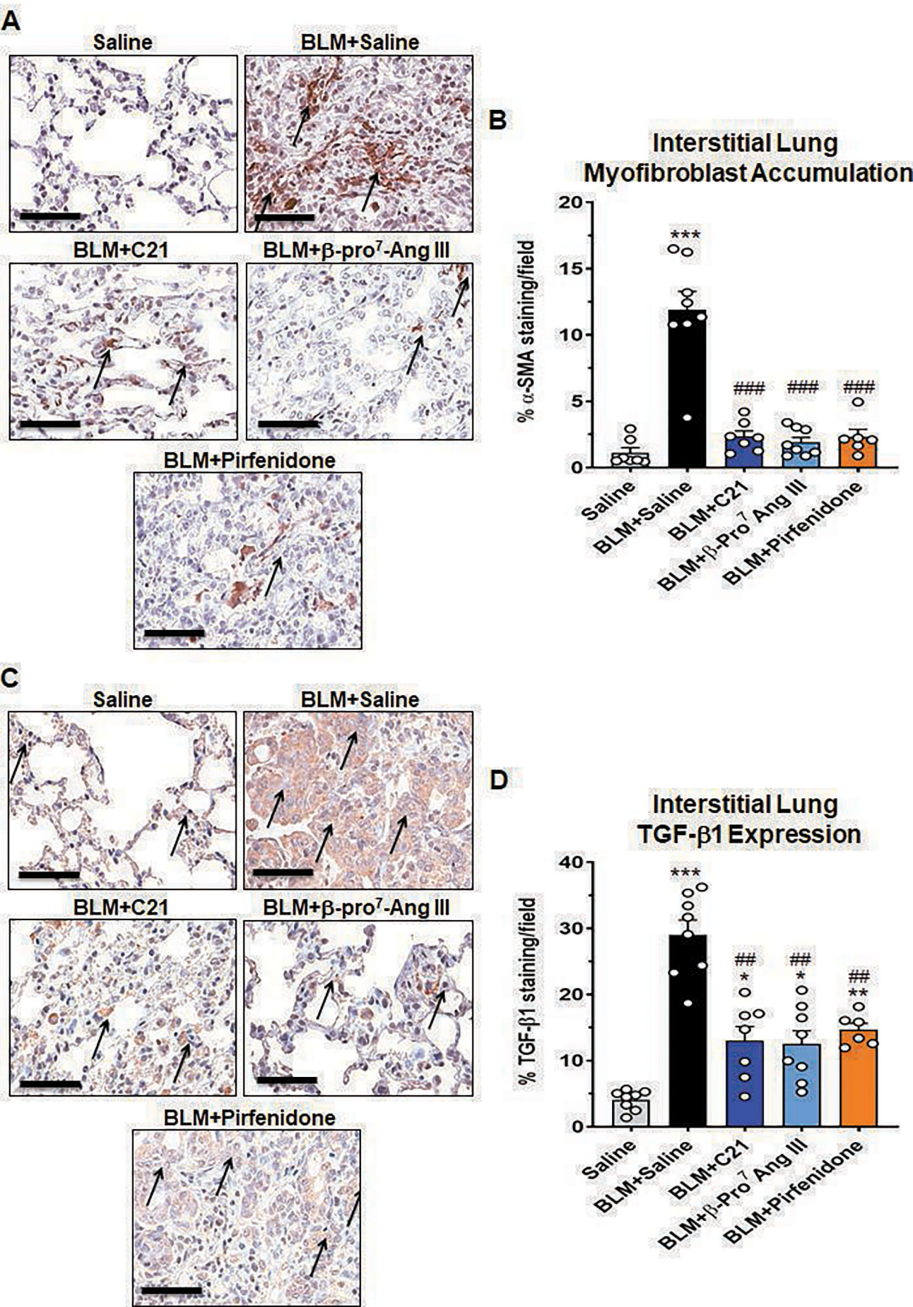
All treatments evaluated attenuated the BLM-induced interstitial lung myofibroblast accumulation and TGF-β1 expression after seven days of treatment. Representative immunohistochemically stained images (at ×400 magnification) show the extent of interstitial α-SMA expression (brown staining; as a measure of myofibroblast accumulation) (**A**) or TGF-β1 expression (brown staining) (**C**) from each group investigated. Scale bar=60 μm. The arrows refer to areas of positive α-SMA (**A**) or TGF-β1 (**C**) staining. Also shown are the mean ± SEM% interstitial α-SMA staining (**B**) or TGF-β1 staining (**D**) per field analyzed; from six to eight non-overlapping fields of view per lung section analyzed; from *n*=6–8 mice per group. The white circles represent the individual data points for each group analyzed. *P< 0.05, **P< 0.01, ***P< 0.001 vs the saline-instilled control group; ^##^ *P*<0.01, ^###^ *P*<0.001 vs the BLM + saline-treated group.

BLM-injured mice treated with SAL presented with a ~9.5-fold increase in interstitial lung myofibroblast accumulation (11.90±1.40% per field analyzed; Figure 3A and B) and ~6-fold increase in interstitial lung TGF-β1 expression (29.03±2.23% per field analyzed; Figure 3C and D) at day-35 post-injury, compared with respective measurements from their SAL-instilled counterparts (interstitial myofibroblast accumulation per field analyzed: 2.23±0.37%; interstitial TGF-β1 expression per field analyzed: 4.07±0.57%; both *P*< 0.001 vs SAL-instilled counterparts). The BLM-induced increase in interstitial lung myofibroblast accumulation was markedly attenuated by all three treatments evaluated (all *P*< 0.001 vs BLM + SAL-treated group; all no different to the SAL-instilled group; Figure 3B) after seven days of treatment, to levels that were no different to that measured from SAL-instilled controls. The BLM-induced increase in interstitial TGF-β1 expression was also significantly attenuated by all treatments evaluated (by 58–66%; C21 treatment: 12.99±2.18%; β-Pro^7^ Ang III treatment: 12.54±2.0%; pirfenidone treatment: 14.67±0.98%; all *P*< 0.01 vs BLM + SAL-treated group), although not fully back to levels measured in SAL-instilled control mice (all *P*<0.05 vs SAL-instilled mice; Figure 3D) after the seven-day treatment period.

### β-Pro^7^ Ang III significantly increased MMP-2 activity in BLM-injured mice after seven days of treatment

**Figure 4:**
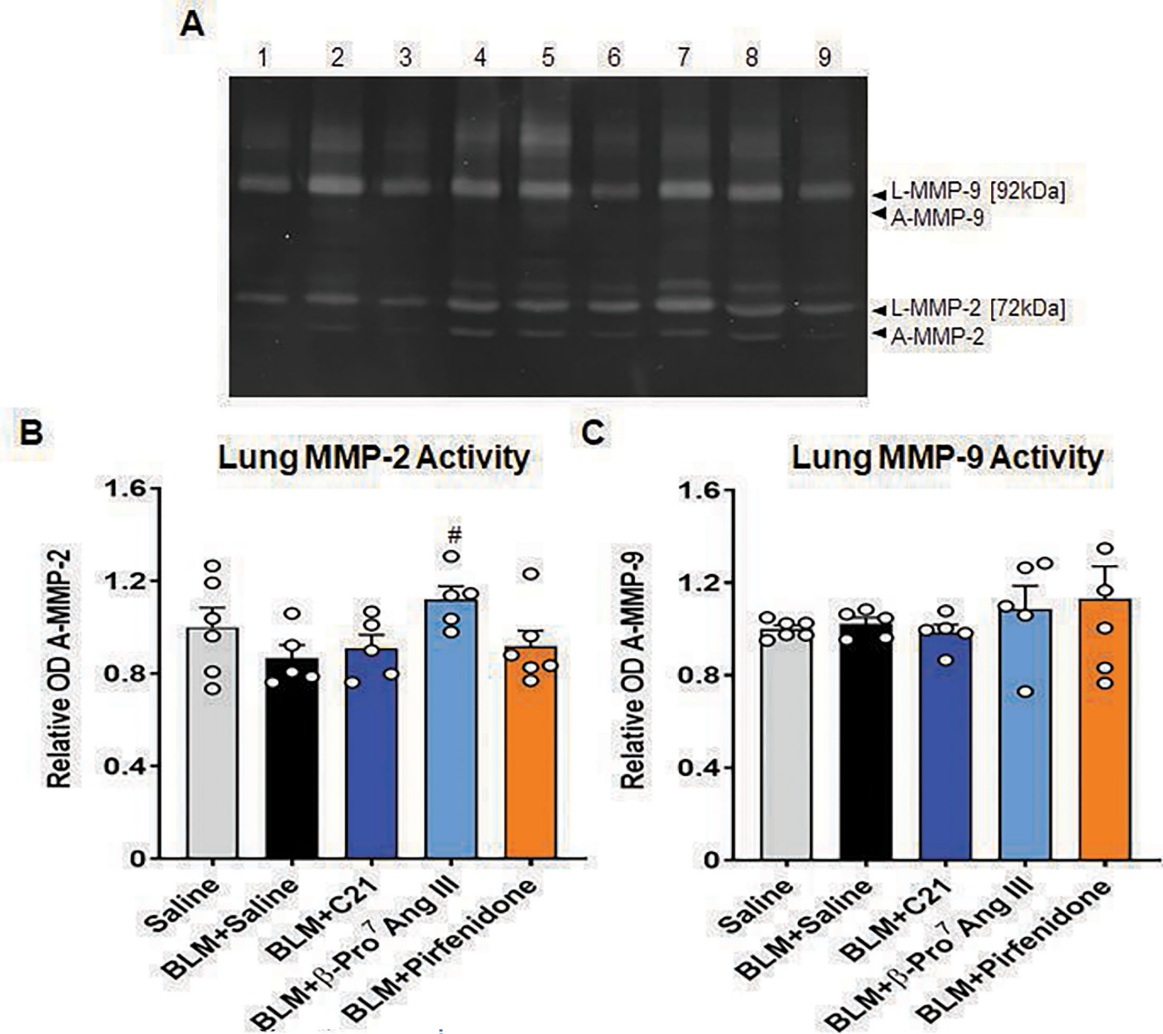
β-Pro^7^ Ang III significantly increased MMP-2 activity in BLM-injured mice after seven days of treatment. A representative gelatin zymography shows the extent of latent (**L**) and active (**A**) MMP-2 (gelatinase A) and MMP-9 (gelatinase B) from each of the groups evaluated. (**A**) Lane 1 shows the levels of these MMPs from a saline-instilled mouse lung protein extract; lanes 2 and 3 show the levels of these MMPs from two separate BLM-instilled + saline-treated mouse lung protein extracts; lanes 4 and 5 show the levels of these MMPs from two separate BLM-instilled + C21-treated mouse lung protein extracts; lanes 6 and 7 show the levels of these MMPs from two separate BLM-instilled+β-Pro^7^ Ang III-treated mouse lung protein extracts; and lanes 8 and 9 show the levels of these MMPs from two separate BLM-instilled + pirfenidone-treated mouse lung protein extracts. An additional *n*=4–5 samples per group were run on separate zymographs, which produced similar results. Also shown are the mean + SEM relative OD lung A-MMP-2 (**B**) or A-MMP-9 (**C**) from each group investigated; from *n*=5–6 mice per group. The white circles represent the individual data points for each group analysed. ^#^ *P*<0.05 vs the BLM + saline-treated group.

BLM-injured mice treated with SAL had a non-significant trend toward having lower levels of A MMP-2 at day-35 post-injury (Figure 4A and B) in the absence of any detectable changes in A MMP-9 levels (Figure 4A and C). β-Pro^7^ Ang III, but not the other treatments evaluated, was able to significantly increase A MMP-2 levels over that measured in SAL-treated BLM-injured mice (*P*< 0.05 vs BLM + SAL-treated group; Figure 4B) after seven days of treatment. On the other hand, neither treatment evaluated affected lung MMP-9 activity after the same treatment period (Figure 4C).

### β-Pro^7^ Ang III significantly restored the BLM-induced loss of dynamic lung compliance after seven days of treatment

**Figure 5:**
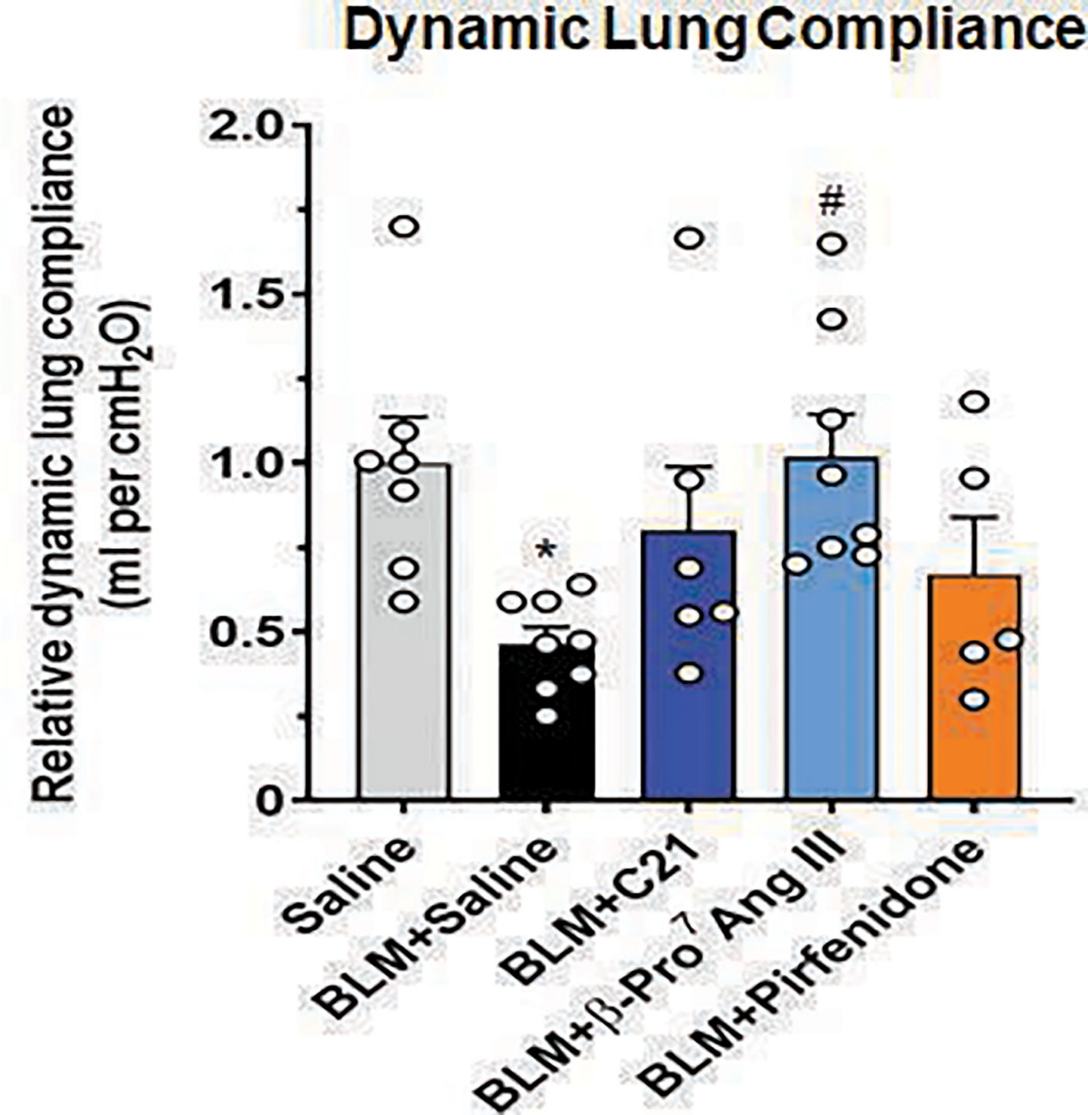
β-Pro^7^ Ang III significantly restored the BLM-induced loss of dynamic lung compliance after seven days of treatment. Shown is the relative lung compliance (measured as ml per cmH_2_O) in each of the groups evaluated, in response to nebulized methacholine (bronchoconstrictor) administered (50 mg/mL); from *n*=6–8 mice per group. The white circles represent the individual data points for each group analyzed. *P< 0.05 vs the saline-instilled control group; ^#^ *P*< 0.05 vs the BLM + saline-treated group.

In line with the increased interstitial lung remodeling and fibrosis that was evident in SAL-treated BLM-instilled mice, these animals underwent a significant loss of dynamic lung compliance in response to the bronchoconstrictor, methacholine (by ~0.55-fold at the highest dose of methacholine tested), compared with that measured in SAL-instilled control mice (*P*< 0.05 vs SAL-instilled control group; Figure 5). This indicated that SAL-treated BLM-instilled mice had stiffened lungs. While this BLM-induced loss of dynamic lung compliance was blunted by seven days of C21 or pirfenidone treatment (to levels that were no longer different from that measured in SAL-instilled control mice), it was only significantly restored by β-Pro^7^ Ang III treatment over the same time period (*P*< 0.05 vs BLM + SAL-treated group; no different to that measured in SAL-instilled control mice; Figure 5).

## Discussion

This study was the first to evaluate and compare the anti-fibrotic effects of the novel AT_2_R agonist, β-Pro^7^ Ang III [30], to the commonly used AT_2_R agonist, C21 [25,51], and the clinically approved TGF-β1 blocker, pirfenidone [12] in a murine model of BLM-induced pulmonary fibrosis. Female BALB/c mice subjected to repeated intranasal BLM instillation presented with significantly increased interstitial lung fibrosis, myofibroblast accumulation, TGF-β1 expression, and a loss of dynamic lung compliance (which is an indicator of lung stiffness) at five weeks post-injury. Given that female BLM-injured BALB/c mice (given two intranasal instillations of BLM, seven days apart) were previously shown to have established fibrosis and a loss of dynamic lung compliance at 3 weeks post-injury [39], this study evaluated the therapeutic effects of the treatments investigated, when given as intervention therapies (i.e. at four weeks post-injury). β-Pro^7^ Ang III exerted equivalent anti-fibrotic effects to that of C21, but broader anti-fibrotic effects compared with pirfenidone after seven days of treatment, and significantly restored the BLM-induced loss of dynamic lung compliance. These anti-fibrotic effects of β-Pro^7^ Ang III were induced through its ability to attenuate interstitial lung TGF-β1 expression and myofibroblast accumulation (which C21 and pirfenidone also achieved), whilst being able to promote MMP-2 activity, which can degrade interstitial collagens [52].

Although dose–response studies were not conducted for each drug tested, the dose of β-Pro^7^ Ang III [33–35] or C21 [53,54] used in this study had demonstrated anti-fibrotic effects in other models of injury-induced fibrosis, whereas the dose of pirfenidone used had prevented [43] or therapeutically reduced [39] BLM-induced lung fibrosis in mice. Indeed, by three different assessments of ECM deposition (Masson’s trichrome staining of interstitial ECM deposition; Ashcroft scoring of lung sections that graded the severity of lung congestion and interstitial ECM deposition; and HistoIndex-scanned analysis of interstitial lung collagen deposition), it was apparent that β-Pro^7^ Ang III or C21 treatment of the BLM model induced equivalent anti-fibrotic effects. While previous chronic studies had found that C21 [53,55] or β-Pro^7^ Ang III [33,35] could therapeutically reduce established collagen deposition after four–eight weeks of treatment, in the current study, a seven-day intervention period was sufficient to attenuate the BLM-induced increase in interstitial collagen deposition. This finding is in keeping with the ability of β-Pro^7^ Ang III to prevent ischemia-reperfusion injury in the kidney after seven days of treatment [34]. Our findings thus demonstrated that the therapeutic targeting of the AT_2_R more broadly attenuated BLM-induced lung ECM deposition compared with the effects of pirfenidone.

The short-term anti-fibrotic effects of β-Pro^7^ Ang III were found to be associated with its ability to significantly reduce interstitial lung TGF-β1 expression levels and myofibroblast accumulation, and increase collagen-degrading MMP-2 activity in BLM-injured mice, which fits with previous studies [16–19]. These findings are in line with reports demonstrating that C21 treatment of BLM-injured rats attenuated right ventricular systolic pressure, lung vessel wall thickness, and lung fibrosis (Ashcroft score and hydroxyproline levels) after 11 days of treatment [25]. These ECM remodeling effects of β-Pro^7^ Ang III were also maintained after four weeks of treatment, when it was administered to high-salt-fed mice [33] or diabetic rats [35]. As C21 attenuated CD68^+^ macrophage infiltration in the lungs of BLM-injured mice [25], while β-Pro^7^ Ang III attenuated F480^+^ or CD68^+^ macrophage infiltration in the kidneys of high-salt-fed mice [33], mice with ischemia-reperfusion injury [34], or diabetic rats [35], it is likely that the C21- or β-Pro^7^ Ang III-induced reduction in lung TGF-β1 expression levels detected in this study was induced by the ability of these compounds to inhibit macrophage infiltration, which is a key cellular source of TGF-β1 production [56].

As inflammation had been found to subside in BLM-injured mice by seven–ten days post-injury [57], the anti-inflammatory effects of the treatments evaluated were not investigated in the current study. However, in previous studies demonstrating the anti-inflammatory effects of C21 [25,58] or β-Pro^7^ Ang III [33–35], these AT_2_R agonists were either given prior to injury onset [58], immediately post-injury [25,34], or three days post-injury [25], or to established disease settings over a four-week treatment period [33,35]. Under these settings, both compounds were found to either prevent or reduce macrophage infiltration [25,33–35] and other measures of lung inflammation (such as interleukin (IL)-6, tumor necrosis factor (TNF)-α, chemokine ligand 2 (also known as monocyte chemoattractant protein 1) and toll-like receptor 4 levels), while promoting anti-inflammatory IL-10 levels in the various models investigated. Hence, it is plausible to suggest that the anti-inflammatory effects of these AT_2_R agonists may have contributed to their observed anti-fibrotic effects, but this needs to be verified in future studies.

The gelatinase (MMP-2)-promoting effects of β-Pro^7^ Ang III may have been linked to AT_2_R activation commonly leading to the promotion of nitric oxide (NO) and cyclic guanosine monophosphate (cGMP) signaling, which are known suppressors of the pro-fibrotic actions of TGF-β1 at the intracellular protein level of suppressor of mothers against decapentaplegic (SMAD)2 [59] and/or SMAD3 [60]. Hence, as TGF-β1, in turn, up-regulates tissue inhibitor of metalloproteinase (TIMP) activity to antagonize the effects of MMPs [61], it is possible that the TGF-β1-inhibitory effects of β-Pro^7^ Ang III via the AT_2_R led to the ability of this highly selective agonist to alter the balance between MMP-2 and its (TIMP) inhibitor, which resulted in its ability to up-regulate MMP-2 activity. Further studies are thus warranted to evaluate the effects of β-Pro^7^ Ang III on NO and cGMP activity within the lung, and its effects on the balance between MMP-2 and its natural inhibitor, TIMP-2. Additionally, studies examining the dose-dependent effects of β-Pro^7^ Ang III over longer treatment periods would provide further insights into its sustained ability to treat IPF and related fibrotic conditions. Notably, the current data fit with preliminary results published that indicate beneficial effects of C21 in IPF [62].

On the other hand, pirfenidone failed to alter the established BLM-stimulated lung ECM/collagen deposition after seven days of treatment, when administered to mice at 28 days post-injury, despite being able to reduce interstitial lung TGF-β1 expression and myofibroblast accumulation within this time frame. Comparably, our previous work had indicated that the same dose of pirfenidone (100 mg/kg/day) significantly reduced interstitial lung TGF-β1 expression, myofibroblast accumulation, and ECM deposition when administered over a seven-day period, to BLM-injured female mice at 21 days post-injury [39]. These findings may be explained by the fact that short-term daily pirfenidone administration was able to therapeutically attenuate the milder BLM-induced interstitial fibrosis that only represented ~6–7% of the fractional lung area stained at day-21 post-injury [39] but could not maintain its effects against the more severe interstitial fibrosis that represented ~20–25% of the fractional lung area stained by day-28 post-injury (Figure 1C). However, 100 mg/kg/day pirfenidone was found to significantly suppress the BLM-induced increase in lung inflammation and fibrosis, when orally administered at the time of injury onset and given three times a day over a six-week period [43], or was able to normalize the BLM-induced interstitial lung fibrosis in mice, when administered at a higher dose (300 mg/kg/day) at ten days post-injury and after four weeks of treatment [63]. Although the therapeutic impact of pirfenidone may vary depending on the timing and duration of its administration, dose administered, and the species it is administered to [64,65], the above-mentioned findings from studies in mice suggest that the efficacy of pirfenidone diminishes as disease severity worsens, requiring the drug to be administered as early as possible and at high doses over long treatment periods (4–6 weeks) to maintain its efficacy. The latter may be somewhat compromised, though, by the well-established side effects of longer term pirfenidone exposure [66,67].

There were a few limitations to this study that should be addressed in the future. First, we did not assess either the effects of C21 or β-Pro^7^ Ang III alone (in uninjured mice), as this study focused on the therapeutic effects of these compounds in the context of lung fibrosis. We did not expect either AT_2_R agonist to induce any effects in healthy mice. This is in line with previous reports demonstrating that the administration of C21 (0.3 mg/kg/day) alone to uninjured male Sprague-Dawley rats did not affect blood pressure, hemodynamic parameters, lung ACE, ACE2, AT_1_R, TGF-β1, or pro-inflammatory cytokine (TNF-α, IL-1β) mRNA levels after two weeks of treatment, but significantly increased lung AT_2_R mRNA expression over this period [51]. Similarly, β-Pro^7^ Ang III (0.1 mg/kg/day) did not affect blood pressure after four weeks of treatment to male high salt (5% sodium chloride)-fed mice that were normotensive [33]. Secondly, we did not combine the effects of C21 or β-Pro^7^ Ang III with the AT_2_R antagonist, PD123319, to confirm the AT_2_R specificity of these compounds in the model investigated, although we had previously reported that the effects of β-Pro^7^ Ang III were blocked by PD123319 [33]. Indeed, it is widely accepted that C21 mediates its effects via the AT_2_R [16–19] and, at the dose (0.3 mg/kg/day) administered, increases lung AT_2_R but not AT_1_R mRNA expression in uninjured and hypertensive rats [51]. Similarly, with >20,000-fold selectivity for the AT_2_R over the AT_1_R, the effects of β-Pro^7^ Ang III were found to be specifically mediated via the AT_2_R [30]. While plasma levels were not measured in the current study, it should be noted that both AT_2_R agonists were infused subcutaneously to avoid any pharmacokinetic influence. Finally, the sex-dependent effects of these compounds were not explored in the model established, which should be followed up in future studies.

## Conclusion

This study was the first to evaluate and compare the anti-fibrotic effects of β-Pro^7^ Ang III with that of C21 or pirfenidone in BLM-injured mice. In doing so, it has highlighted the broader anti-fibrotic effects that were induced by activation of the AT_2_R in comparison with that provided by the FDA-approved IPF treatment, pirfenidone. Moreover, it has demonstrated that the novel AT_2_R agonist, β-Pro^7^ Ang III, was able to attenuate lung fibrosis through its ability to ameliorate the pro-fibrotic impact of TGF-β1 on myofibroblast accumulation and ECM deposition, while being able to promote collagen-degrading MMP-2 activity after as little as seven days of treatment. These findings endorsed the need for developing high-affinity AT_2_R agonists, as AT_2_R stimulation alone provided broader anti-fibrotic efficacy over that produced by pirfenidone. Based on the current clinical evaluation of C21, it is envisaged that these more selective AT_2_R agonists will follow suit and be evaluated for their clinical application as treatments for fibrotic diseases.

**Clinical Perspectives**

- Background: The angiotensin II type 2 receptor (AT_2_R) agonist, Compound 21 (C21), has well-established anti-fibrotic and organ-protective actions that have led to its clinical evaluation for various indications, including COVID-19, systemic sclerosis, and idiopathic pulmonary fibrosis (IPF). However, C21 can induce off-target effects when administered at high concentrations.
- Summary: This study compared the anti-fibrotic efficacy of the newly developed AT_2_R ligand, β-Pro^7^ Ang III, which has >20,000-fold selectivity for the AT_2_R over the AT_1_R, with the effects of C21 or the FDA-approved medication for treating IPF, pirfenidone, in bleomycin-injured mice with established pulmonary fibrosis. β-Pro^7^ Ang III was found to have equivalent anti-fibrotic effects, including a greater ability to promote the collagen-degrading enzyme, matrix metalloproteinase-2, compared with C21. Notably, though, the targeting of the AT_2_R with either β-Pro^7^ Ang III or C21 more broadly attenuated lung fibrosis in the model established compared with pirfenidone.
- Potential significance of the results to human health and disease: The AT_2_R, which is up-regulated in diseased states, is an attractive target for the therapeutic targeting of fibrosis. Hence, the development and evaluation of more selective AT_2_R ligands, which provoke the organ-protective effects of AT_2_R activation in the absence of any off-target effects via other receptors, will likely lead to safer therapies that can be clinically evaluated for the treatment of human fibrotic diseases such as IPF.

## Data Availability

The data supporting the findings from this study are available within the manuscript. Any remaining raw data are archived in a Monash University repository and will be available from the corresponding author upon reasonable request.

## Abbreviations

A: active
AT_1_R: angiotensin type 1 receptor
AT_2_R: angiotensin type 2 receptor
BLM: bleomycin
C21: Compound 21
ECM: extracellular matrix
FDA: Food and Drug Administration (of the USA)
GPCR: G protein-coupled receptor
IHC: immunohistochemical
IL: interleukin
IPF: idiopathic pulmonary fibrosis
MMP: matrix metalloproteinase
NO: nitric oxide
OD: optical density
SAL: saline
SHG: second harmonics generation
SMA: smooth muscle actin
SMAD: suppressor of mothers against decapentaplegic
TGF-β1: transforming growth factor-β1
TIMP: tissue inhibitor of metalloproteinase
b-pro^7^-Ang III: b-pro^7^-Angiotensin III
cGMP: cyclic guanosine monophosphate
i.n.: intranasally
